# A live-cell platform to isolate phenotypically defined subpopulations for spatial multi-omic profiling

**DOI:** 10.1371/journal.pone.0292554

**Published:** 2023-10-11

**Authors:** Tala O. Khatib, Angelica M. Amanso, Christina M. Knippler, Brian Pedro, Emily R. Summerbell, Najdat M. Zohbi, Jessica M. Konen, Janna K. Mouw, Adam I. Marcus

**Affiliations:** 1 Department of Hematology and Medical Oncology, Emory University School of Medicine, Atlanta, Georgia, United States of America; 2 Winship Cancer Institute of Emory University, Atlanta, Georgia, United States of America; 3 Graduate Program in Biochemistry, Cell, and Developmental Biology, Emory University, Atlanta, Georgia, United States of America; 4 Department of Pathology, Johns Hopkins School of Medicine, Baltimore, Maryland, United States of America; 5 Office of Intramural Training and Education, The National Institutes of Health, Bethesda, Maryland, United States of America; 6 Graduate Medical Education, Piedmont Macon Medical, Macon, Georgia, United States of America; Brunel University, UNITED KINGDOM

## Abstract

Numerous techniques have been employed to deconstruct the heterogeneity observed in normal and diseased cellular populations, including single cell RNA sequencing, *in situ* hybridization, and flow cytometry. While these approaches have revolutionized our understanding of heterogeneity, in isolation they cannot correlate phenotypic information within a physiologically relevant live-cell state with molecular profiles. This inability to integrate a live-cell phenotype—such as invasiveness, cell:cell interactions, and changes in spatial positioning—with multi-omic data creates a gap in understanding cellular heterogeneity. We sought to address this gap by employing lab technologies to design a detailed protocol, termed Spatiotemporal Genomic and Cellular Analysis (SaGA), for the precise imaging-based selection, isolation, and expansion of phenotypically distinct live cells. This protocol requires cells expressing a photoconvertible fluorescent protein and employs live cell confocal microscopy to photoconvert a user-defined single cell or set of cells displaying a phenotype of interest. The total population is then extracted from its microenvironment, and the optically highlighted cells are isolated using fluorescence activated cell sorting. SaGA-isolated cells can then be subjected to multi-omics analysis or cellular propagation for *in vitro* or *in vivo* studies. This protocol can be applied to a variety of conditions, creating protocol flexibility for user-specific research interests. The SaGA technique can be accomplished in one workday by non-specialists and results in a phenotypically defined cellular subpopulations for integration with multi-omics techniques. We envision this approach providing multi-dimensional datasets exploring the relationship between live cell phenotypes and multi-omic heterogeneity within normal and diseased cellular populations.

## Introduction

Cellular heterogeneity underlies all biological systems. Heterogeneity exists across the varying stages of development, differentiation, and disease over length scales from DNA to organism [1–3]. This cellular heterogeneity emerges as a result of epigenetic, transcriptional, and post-translational diversity within and between populations [4, 5]. These heterogeneous populations cooperate to maintain biological homeostasis, often providing a selective advantage by enabling a heightened response to stimuli, microenvironment, or selective pressures [6–8]. Additionally, pathological states emerge and progress under the influence of vast heterogeneity providing diseases, such as cancer, a myriad of potential mechanisms for therapeutic evasion, escape of immune surveillance, and relapse [9–11]. Ultimately, an effective understanding of the temporal progression for any normal or diseased biological system requires consideration of the interplay and cooperation between genetically, epigenetically, and phenotypically diverse cellular subpopulations.

Heterogeneous cellular populations orchestrate diverse phenotypic responses that can be imaged over space and time. However, technologies capable of deriving multi-omic analysis from live, spatiotemporally defined cellular populations are limited. Here, we address this gap in technology by applying live-cell microscopy to explore phenotypic cellular diversity and identify distinct subpopulations across cellular landscapes. This protocol takes a phenotype-driven, live-cell imaging approach to link historical cellular behavior with multi-omic and molecular information. We describe in detail how to exploit accessible lab technologies to isolate user-defined live cells with minimal space and time limitations.

### Development of SaGA

Global multi-omics approaches are commonly utilized to test biological hypotheses, where bulk -*omics* techniques (*e*.*g*., proteomics via mass spectrometry, RNA- and DNA-sequencing) are readily available and cost-effective [12, 13]. For example, these technologies have driven critical discoveries in cancer research including insight into the regulation of onco- genes and proteins [14–16]. One notable drawback to “homogenizing” bulk multi-omics approaches is the inability to discern contributions from heterogeneous and rare subpopulations within each sample. More recent advances in single cell multi-omics provide insight toward resolving the distinct landscapes of subpopulations within a single population of cells; however, these approaches typically do not integrate phenotypic information within a physiologically relevant, live-cell state. Similarly, spatial multi- omics is a powerful tool to collect detailed molecular characterization of tissue while preserving spatial context, however samples are fixed and therefore cannot be propagated for further analysis [17].

**Fig 1:**
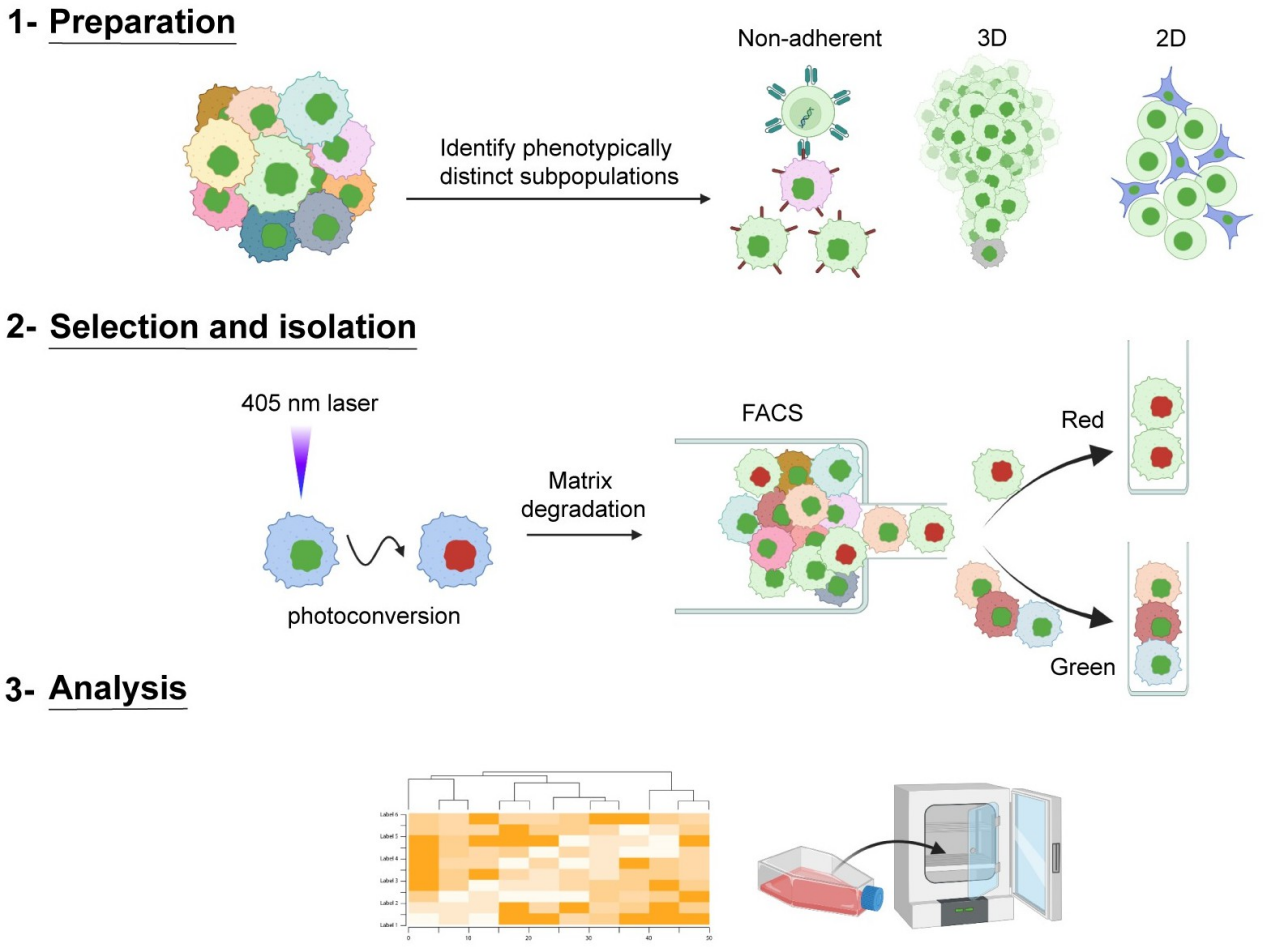
SaGA schematic to isolate distinct cell(s) based upon live, user-defined phenotypic criteria. Schematic showing three broad steps of SaGA: 1) Preparation, 2) Selection and isolation, and 3) Analysis. SaGA can be applied to a variety of cell conditions, such as non-adherent, 3-dimensional (3D), and 2-dimensional (2D), for selection, isolation, and analysis of live subpopulations within a parental population. Cells stably expressing a photoconvertible tag can be precisely photoconverted (from green to red) based upon live, user-defined, phenotypic criteria. These red photoconverted cells are then isolated utilizing fluorescence activated cell sorting (FACS) for multi-omic analysis and/or cell cultivation for long-term *in vitro* and *in vivo* analyses. Created with Biorender.com.

Tumor subpopulations incur distinct genomic and epigenetic profiles through selective pressures, increased genomic instability, and various degrees of entropy; these distinct subpopulations may drive unique invasive potentials and proliferative capacities, where cellular subpopulations cooperate to drive efficacious tumor progression and metastatic disease [11, 18–26]. Despite our knowledge of this cellular heterogeneity, the mechanisms underlying pack formation, function, and impact on cancer progression are largely unknown. To probe subpopulation heterogeneity and its role in collective invasion, we sought to develop an image-guided technique that allows for precise *in situ* selection, isolation, and expansion of phenotypically distinct, live cells and populations during 3D invasion [27]. By combining 3D cell culture with accessible lab technologies—including live-cell confocal microscopy and fluorescence-activated cell sorting (FACS)—the Spatiotemporal Genomic and Cellular Analysis (SaGA) technique allows for the optical marking of single live cells (or collections of cells) within a defined region of interest using a photoconvertible tag, Dendra2 (Fig 1). After photoconversion, the cells can be further observed *in situ* or removed from their environment and flow-sorted for a myriad of downstream analyses including, genotypic and phenotypic stability and/or flexibility of clones and subpopulations (Table 1). Alternatively, these extracted cells can be immediately processed for single or bulk cell multi-omics applications.

**Table 1:** Example downstream applications of SaGA-isolated subpopulations.

Experimental approach		Application	Potential outcome
Immediate isolation	*In vitro*	Cell lysis for immediate contents extraction (i.e., protein, RNA, DNA, and ribosomes)	Targeted transient expression profiling via immunoblotting, qPCR, etc.
			Unbiased transient expression profiling via ATACseq, RNAseq, Riboseq, etc.
Long-term cultivation *in vitro*	*In vitro*	Cell behavior, signaling, etc.	Stable phenotype identification, stable subpopulation generation, determination of cooperative phenotype between subpopulations, targeted expression profiling and unbiased multi-omic analysis.
	*In vivo*	Introduction to model organism	Stable phenotype identification, determination of cooperative phenotype between subpopulations, targeted expression profiling and unbiased multi-omic analysis.

Combining SaGA and multiple non-small cell lung cancer (NSCLC) lines, cell(s) were photoconverted based upon their spatial positioning within the collectively invading pack to isolate leader (front of the pack) and follower (trailing behind leaders) subpopulations [27]. To assess phenotypic stability over time, cells were sorted for propagation and long-term phenotypic and transcriptomic analysis [27–31]. Epigenetic differences were assessed by performing a DNA methylation array, where cells were sorted for immediate DNA extraction and epigenetic analysis [27, 29]. Taken together, these multi-omic results corroborate leader and follower spatial localization, providing, for the first time, a detailed mechanistic understanding of cellular positioning within the collective invasion pack.

### Application of SaGA

The SaGA approach integrates standard laboratory practices to ask fundamental and clinically relevant questions about the mechanistic underpinnings of population heterogeneity. Prior to performing live-cell imaging, SaGA is flexible in experimental design and, therefore, adaptable toward a multitude of research-specific interests. Researchers can readily adopt this protocol with limited experience in confocal imaging or flow cytometry and may find applications in a range of fields including neuroscience or developmental biology [32–34].

Using the H1299 NSCLC line, we found that leaders and followers isolated during 3D collective invasion have distinct genotypic, epigenetic, and phenotypic differences, and are phenotypically stable over many passages [27–31]. By mapping our bulk RNA sequencing results to the human reference genome Hg19 (GRCh37) and various filtering steps, we identified 14 distinct missense mutations between leaders and followers [31]. Similarly, epigenetic analysis via DNA methylation array featured global epigenetic rewiring in leaders compared to followers [29]. These results indicate that our NSCLC spatial localization is a coordinated patterning driven by genomic and epigenetic cellular profiles. At the RNA and protein levels, we found that underlying differences in their filopodia dynamics driven by a Jag1-Myo10 signaling axis further contribute to the stark differences in invasive capacities of the leaders and followers [29–31]. Similarly, invasive chains harbor metabolic heterogeneity, in which trailing followers are highly glycolytic and leaders depend upon mitochondrial respiration [28]. Taken together, these data highlight the ability to use phenotypic heterogeneity to decipher the genomic, epigenetic, and phenotypic underpinnings of tumor cell heterogeneity, and support the application of SaGA to investigate population heterogeneity.

Beyond phenotypic positioning within a collective invasion pack in NSCLC, SaGA can be applied to any image-able phenotype for selective enrichment. Applications include selection of cells based upon the sub-cellular localization of a protein of interest, proliferation rates, drug resistance, homo- or hetero-typic cellular interactions, and morphological changes due to differential cellular environments. During tissue and embryonic morphogenesis, complex architectural and temporal patterns of protein expression emerge. In this instance, SaGA can be readily utilized to answer questions governing cell fate decision making. Similarly, neurological diseases also highlight intercellular heterogeneity [35]. One example is Alzheimer’s disease in which microglia, specialized tissue-resident macrophages in the central nervous system, have distinct localization patterning where SaGA can be utilized isolate microglia based upon their localization [36]. In sum, SaGA provides a powerful platform for deconstructing live-cell phenotypic heterogeneity within any image-able, heterogenous cell population.

### Experimental design and limitations

**Fig 2:**
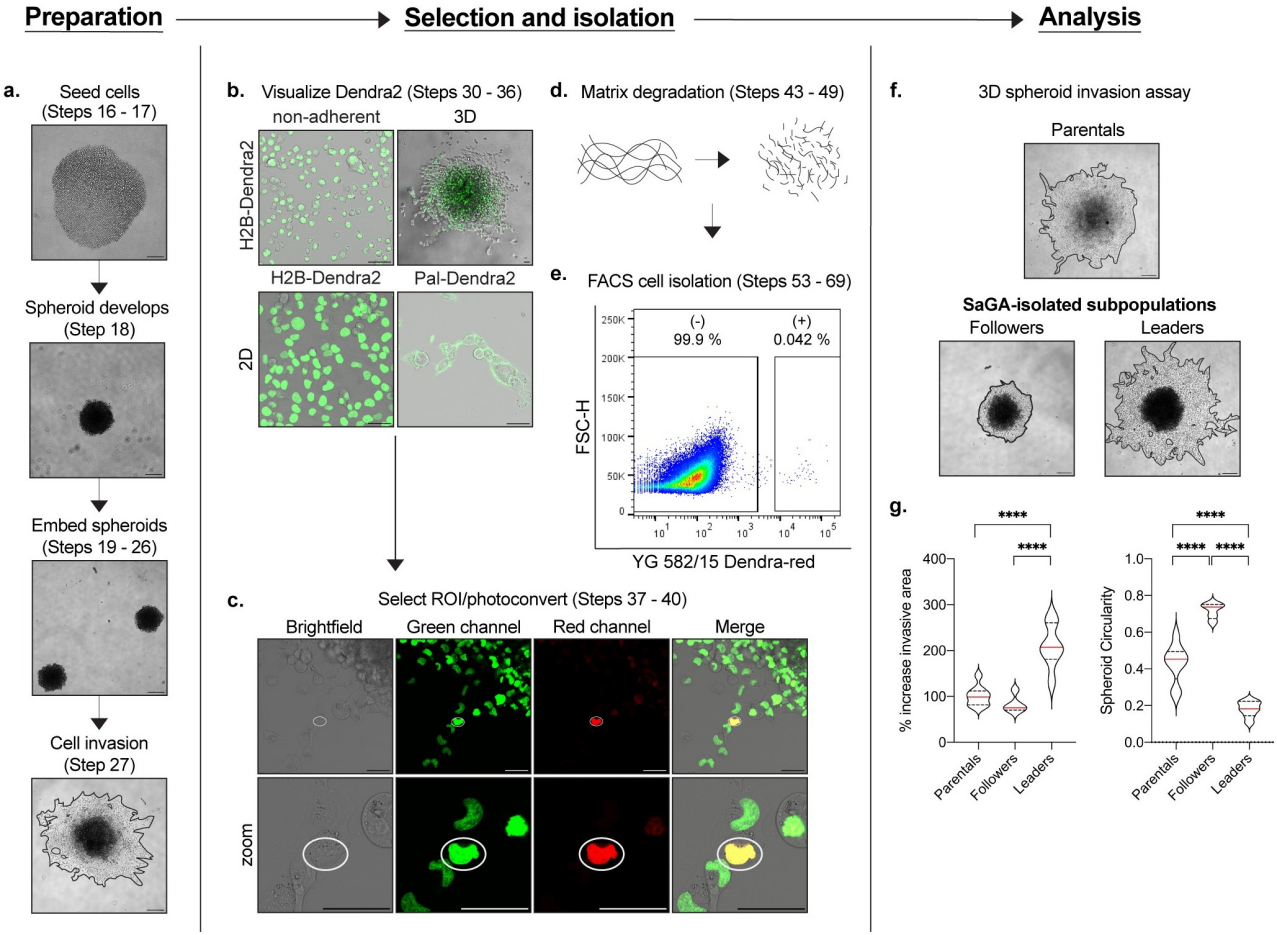
SaGA workflow. Each panel provides an example of a major component of SaGA: Preparation, Selection and isolation, and Analysis. **a.** 3D spheroid invasion assay set-up beginning with spheroid formation in a low adherence 96-well plate to embedment and invasion in recombinant basement membrane. Scale bar, 250 μm. **b.** Dendra2 visualization under non-adherent, 3D and 2D conditions. 2D conditions are shown utilizing both nuclear- (H2B-Dendra2) and membrane- (Pal-Dendra2) localized protein tags. Scale bar, 50 μm. **c.** Defining a region of interest (ROI) (white circle) for cell selection and photoconversion. Scale bar, 50 μm. **d.** Matrix degradation in 3D conditions utilizing collagenase/dispase cocktail. **e.** FACS plot showing non-photoconverted (-) and photoconverted (+) cells. **f.** 3D spheroid invasion assay with H1299 parental population and SaGA-isolated leader and follower subpopulations. Scale bar, 250 μm. **g.** Invasive area and spheroid circularity quantification. *p < 0.05 by one-way ANOVA with Tukey’s multiple comparisons test.

**Fig 3:**
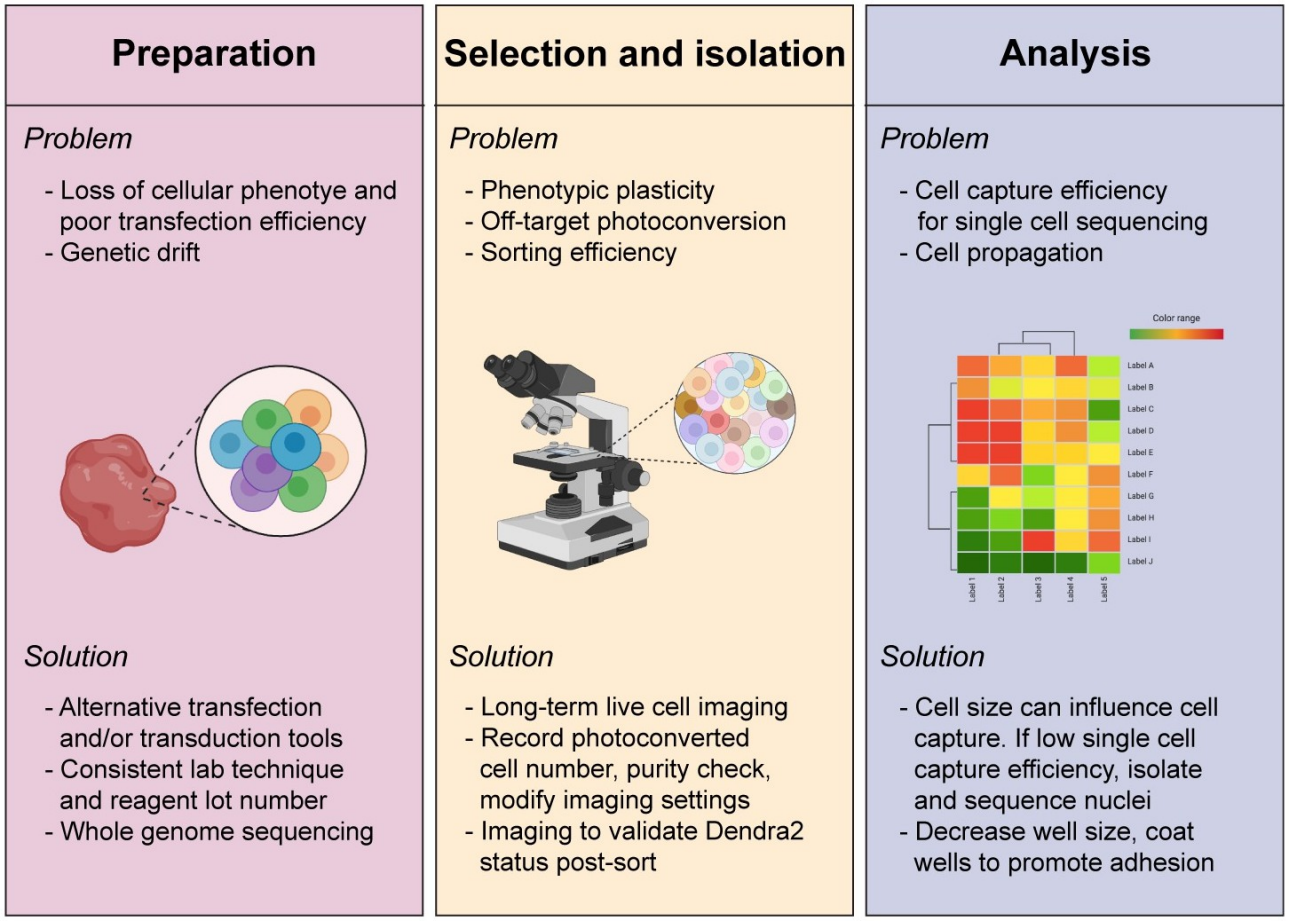
Potential loss of heterogeneity and error sources and measures to minimize them. Cellular loss of heterogeneity can occur during sample preparation, selection and isolation, and analysis. Listed is each major stage of SaGA with potential problems (bulleted above image within each panel) that can occur and respective potential solutions (bulleted below image within each panel). Graphical images created with Biorender.com.

Here, we elucidate key steps in the experimental strategy of SaGA, a method to isolate and evaluate the molecular dependencies of any image-able, phenotypically distinct cell subpopulation in live cell microscopy. Broadly, implementing SaGA requires a tissue culture grade facility, a laser scanning confocal microscope with a photoconversion regime, molecular biology approaches suitable for genetic manipulations, and a fluorescence activated cell sorter (FACS). Overall, the protocol includes cellular introduction with a photoconvertible tag, live cell imaging to photoconvert the region of interest (ROI), FACS to isolate the cell subpopulation of interest, and downstream multi-dimensional analysis (Table 1, Fig 2). These techniques are accompanied by a set of technical limitations, highlighting the importance of minimizing experimental bias and maintaining initial population heterogeneity (Fig 3). In the following section, we discuss how to best accommodate these limitations to facilitate and maintain population heterogeneity (Fig 3).

#### Choice of fluorescent protein

Fluorescent tags have evolved over decades, from green fluorescent protein (GFP), discovered in 1962, to photoconvertible fluorescent proteins (PCFPs), discovered in 2002; PCFPs are characterized by their ability to switch emission spectra upon illumination with light at a specific wavelength and intensity, thereby allowing precise optical labeling and tracking of protein or cell dynamics [37–39]. Many PCFPs have been designed, including the green-to-red Dendra2 and mEos2 proteins, the orange-to-far-red PSmOrange protein, and the cyan-to-green PS-CFP2 protein [40–44]. These fluorescent molecules can also be targeted to distinct cellular regions through additional sequence modifications, allowing for precise photoconversion of sub-cellular structures such as the plasma membrane, nuclei, or mitochondria.

Choice of PCFP requires careful consideration of the experimental question, model and PCFP dynamics, and potential limitations. Previously published studies in our lab utilized SaGA to isolate cells based upon their location within a collective invasion pack. As such, to better distinguish the physical positioning between and amongst cells, we chose to use either a histone H2B-tagged Dendra2 (which localizes to the nucleus) or a palmitoylated Dendra2 (which localizes to lipid rafts, including those in the plasma membrane) for our experimentation. The Dendra2 PCFP is an engineered Kaede-like fluorescent monomeric protein with a light-driven covalent modification that results in an irreversible photoconversion [45, 46]. Dendra2 can be initially excited at 490 nm to fluoresce in the GFP-like green fluorescent state (emission peak at 507 nm). Upon user-defined exposure to UV-violet or blue light, Dendra2 irreversibly photoconverts to a red fluorescent state (excitation/emission peaks at 553/573 nm) [37, 39, 47]. This PCFP has flexibility in that photoconversion can occur with either UV-violet (360–420 nm) or blue (460–500 nm) light excitement.

#### Cell transfection and transduction

Introducing genetic material into a cell can be performed stably or transiently with a myriad of well-established biological, chemical, and physical methods [48–50]. While definitions vary in the literature, for clarity and the purposes of this protocol, we define transfection as the introduction of genetic material into a cell via non-viral methods, and transduction as the introduction via viral methods. The technique chosen depends upon the cell model and the experimental requirements. Ensuring adequate brightness, expression level, and relatively homogenous PCFP expression is a key first step in an experimental design centering around maintaining representative phenotypic heterogeneity.

Our laboratory has used multiple techniques for stably introducing Dendra2 into various cell lines and populations. For our H1299 NSCLC (with pal-Dendra2) and myeloma (using H2B-Dendra2) lines, the cells were stably transduced with their respective Dendra2 plasmids using standard 2^nd^ generation lentiviral transduction methodologies; more extensive protocol information can be found online at Addgene and the Trono lab websites [51, 52]. For our 4T1 mouse mammary carcinoma line, we introduced H2B-Dendra2 via a non-viral DNA transposon system (Sleeping Beauty) for stable integration into the genome; for cell populations and subpopulations resistant to viral transduction, a transposon system allows for the stable expression using any non-viral transfection delivery method [53, 54]. After stable introduction of Dendra2, sort Dendra2 positive cells with homogeneous Dendra2 expression using FACS. Of note, the ectopic expression of any fluorescently labeled protein can result in an artificially high “overexpression” that can lead to unintended off-target activity. Additionally, depending on the method of plasmid introduction, cell lines can have different transfection or transduction efficiencies across individual subpopulations within the parental populations, leading to a loss of heterogeneity or a shift in subpopulation percentages within the overall parental population. Another approach is introducing a transient cell permeable dye, such as (E)-3/ (Z)-3 Mitochondrial dye, to the cellular system [55]. The (E)-3/ (Z)-3 Mitochondrial dye is a noncytotoxic mitochondria-specific dye that circumvents cellular loss of heterogeneity that can be induced by stable integration techniques. Regardless, less obvious cellular functions may not be preserved with the addition of the exogenous element. Therefore, for each cell line of interest, it is important to confirm its heterogeneity characteristics and phenotypes of interest after PCFP introduction. For our SaGA experiments, we confirmed no significant difference in invasive properties, cellular circularity and morphology, subpopulation percentages and cell phenotype. Further information and additional techniques for PCFP introduction can be found in the literature [48, 56, 57].

#### Tissue culture conditions

**Fig 4:**
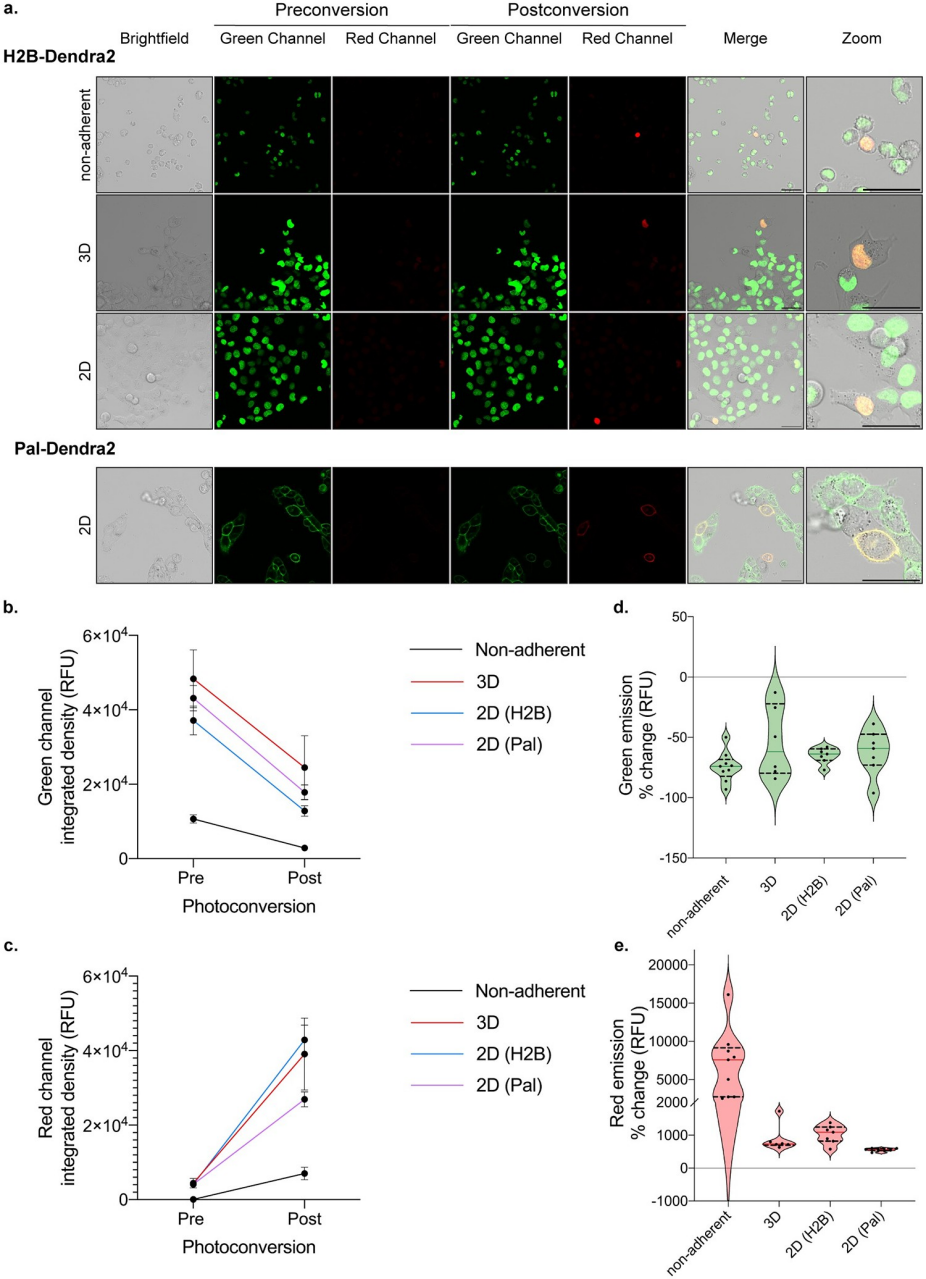
Example photoconversion in different cell culture conditions. **a.** Cells stably expressing a photoconvertible tag (ex: H2B-Dendra2, Pal-Dendra2) can be prepared under non-adherent, 3D, or 2D experimental conditions which illicit distinct and imageable cellular response for photoconversion. Non-adherent conditions were performed with RPMI8226 myeloma cells; H1299 lung cancer cells were used for all other conditions. Scale bar, 50 μm. **b, c.** Integrated density (relative fluorescence units) quantification of 6 or more cells pre- and post- photoconversion in the green (b) and red (c) channels, emission peaks, 507 nm, and 573 nm, respectively. **d, e.** Quantification of integrated density percent change of 6 or more cells pre- and post- photoconversion in the green (d) and red (e) channels.

Three common culture conditions are often utilized to assess cell phenotype *in vitro*—non-adherent, 2D and 3D culture. Depending on the specific cell type and/or experimental question, SaGA can and has been successfully used in all three settings (Fig 4A). SaGA can be implemented under non-adherent culture to determine a variety of biological phenomena including differential cell responses to a treatment (i.e., cytokine, growth factor, drug, starve/stimulation) or in heterotypic cell mixing experiments. For example, glioma cells lose their ability to grow diffusely in the brain when grown as adherent cells and, therefore, passage and analyses in suspension under non-adherent conditions provide an *in vitro* environment conducive to modeling that particular *in situ* behavior [58]. Traditional 2D monolayer (including growth on standard tissue culture plastic dishes) is a common approach to exploring a range of cell biology questions and provides a simpler environment to implement SaGA. Additionally, SaGA can be combined with 3D culture techniques with physiologically and pathologically relevant extracellular matrices (ECMs). 3D culturing techniques allow for active matrix and structural cell remodeling through a “dynamic reciprocity” between cell and environment that has been shown to more faithfully recapitulate tissue specific function compared to 2D conditions [59, 60]. In our laboratory, SaGA has been utilized to observe invasive phenotypes in a variety of ECMs, with different cell lines, each having their own distinct invasion phenotypes (Tables 2 and 3, Fig 2A and 2F).

**Table 2:** Parameters for spheroid formation.

Parameters	Optimization	Examples	Steps
Seeding density	Spheroid density depends upon cell size, shape, morphology, and rate of proliferation. Various densities should be screened to achieve ~ 500 μm in spheroid diameter upon embedding into matrix. Importantly, the larger the cell number, the larger the spheroid, the greater oxygen differential between the external cells and the cells internal to the 3D structures.	3000 cells/well (H1299, 4T1) 1000 cells/well (A375)	Steps 16–18
Nanoparticle contamination	Sterile 96-well plates and/or sterile pipet tips are often contaminated with sterilized nanoparticles that can become embedded within a spheroid and deform its shape. 1.5X spheroids are created to account for unusable spheroids.		Steps 16–25
Cell adherence	Heterogeneous cells can express distinct adherence junction profiles to regulate cell—cell junctions and cell—matrix adhesion/interactions. Centrifugating 96-well plate places cells in the center of the well near one another to promote cell—cell junction formation. Upon spheroid formation, different matrices can be screened to determine the ability for cell—matrix adhesion formation and interactions.	rBM (H1299, A375) Collagen I (4T1)	Step 18
Time	After centrifugation, spheroid formation requires a 24 h or more incubation time. Cells can be screened to determine optimal incubation time to maintain both spheroid integrity during the embedding process and cell viability after.	72 h (H1299, 4T1, A375)	Step 18

**Table 3:** 3D spheroid invasive area and circularity quantification.

It is important to ensure that spheroid invasion dynamics remain largely unaffected when cells are transduced with photoconvertible tag. (The same principles can be applied to confirm no off-target effects from tag in user assay of choice)
**Procedure**—Timing 3 days (imaging and spheroid invasion), 1 h (imaging analysis)
1. Establish and embed spheroids with and without photoconvertible tag (Steps 16–26).
2. Image spheroid on day 0, day 1, day 2 using Compound light microscope at 4X (Step 27).
3. Transfer imaging data and open FIJI software (or other software of your choice).
4. Set up analysis tools to determine object circularity and surface area. Use the ‘draw’ icon to create an outline of each spheroid (including invading cells).
5. Calculate and surface area for each experimental group and export data to excel to determine standard deviation between spheroid technical replicates.
6. Compare results to determine statistically distinct differences in invasive area or circularity between naïve cells and those transduced with photoconvertible tag.

#### Live cell imaging and photoconversion

We conducted live cell imaging on a Leica TCS SP8 inverted point scanning confocal microscope equipped with a stage top incubator to maintain cell culture conditions while imaging. This microscope provides flexibility to modify laser intensity settings and includes a module for fluorescence recovery after photobleaching (FRAP, used for photoconversion here), where short laser pulses in a spatially defined region produce high energy light to induce spectral changes. FRAP has a longstanding history of being utilized to dissect protein dynamics and molecular diffusion, and many modern microscopes are programmed to include a pre-existing FRAP option [61–63]. Photoconversion uses a similar microscopy setup as FRAP, with the addition of a second (post-conversion) emission detection step; as such, the FRAP module may often be adaptable to the photoconversion step within SaGA. Within this module, a user can define a precise region of interest (ROI) that can vary from sub-cellular to multi-cellular in area (Fig 4A). Microscopes with similar point scanning confocal, live cell capabilities, and appropriate laser lines, along with scanning-photoconversion modes, can be used to perform SaGA.

Dendra2 utilizes three laser lines for excitation and photoconversion: 405 nm or 488 nm (implement photoconversion), 488 nm (excitation pre-photoconversion, detection 490–550 nm), and 543-, 561-, or 568 nm (excitation post-photoconversion, detection 570–670 nm). Importantly, any additional small molecule or antibody dual-labeling is limited to the far-red spectrum [46]. Dendra2 photoconversion results in an approximate 50% decrease in the green channel and greater than 500% increase in the red channel, independent of culture conditions (Fig 4B–4E). Further, photoconversion of Dendra2 (Dendra2-green) is irreversible and after 14 hours we still observe photoactivated Dendra2 red fluorescent signal (Dendra2-red) utilizing our current optical settings. If the experimental procedure requires more than 24 hours between photoconversion and fluorescence activated cell sorting, the PS-CFP2 PCFP has been shown to remain stable for 48 hours and can be used as an alternative option [64].

**Fig 5:**
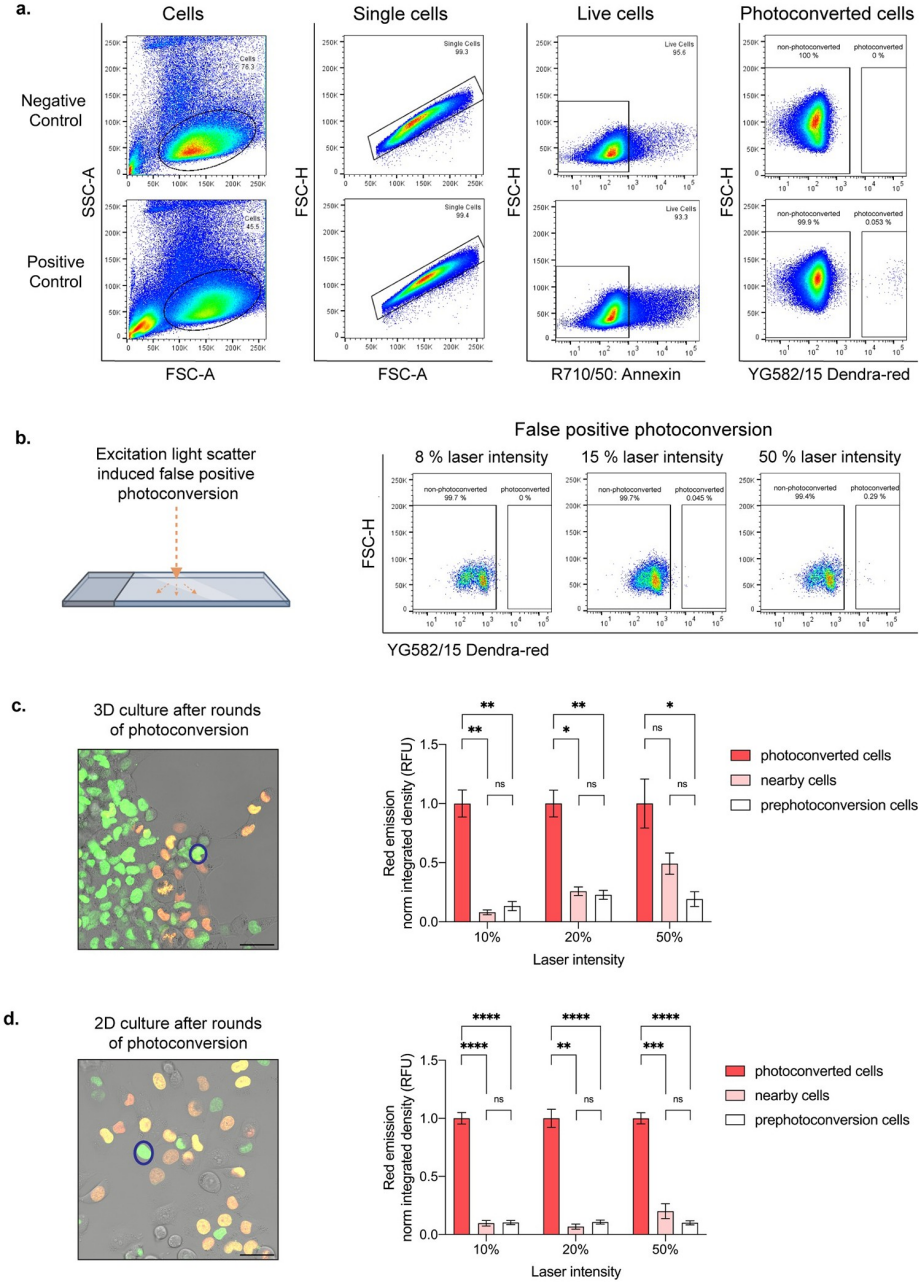
I cell selection and isolation optimization. **a.** Flow plots illustrating stepwise isolation of live photoconverted cells. 8% 405 nm laser line intensity utilized in positive control. **b.** False positive photoconverted cells due to light reflection off the glass plate at varying photoconversion laser intensities at 405 nm. **c.** Representative merged image showing photoconversion of multiple cells (orange and yellow cells) in 3D, where intensity change is measured in a neighboring, non-photoconverted cell (representative nearby cell circled in blue). Quantification of 6 or more cells showing fold change of normalized red emission after rounds of photoconversion are complete. **d.** Representative merged image showing photoconversion in multiple cells (orange and yellow cells) in 2D, where intensity change is measured in a neighboring, non-photoconverted cell (representative nearby cell circled in blue). Quantification of 6 or more cells showing fold change of normalized red emission after rounds of photoconversion are complete. *p < 0.05 by one-way ANOVA with Tukey’s multiple comparisons test. Scale bar, 50 μm.

Insufficient photoconversion due to inadequate excitation light and imaging parameters can yield poor photoconversion efficiency. Notably, low photoconversion efficiency can lead to poor separation between non-photoconverted cells and photoconverted cells during FACS, which can compromise sorted cell purity. Conversely, photobleaching or phototoxicity may result from excessive laser power or excitation time. Photoconversion using illumination with the 405 nm laser line may be best resolved in short pulses with low laser intensity to avoid DNA damage (as UV damage disrupts nuclei division [65]). Alternatively, the 488 nm laser can be applied for more continuous pulses at low or moderate intensity; for this modification, the decreased efficiency of the photoconversion stimulated by a 488 nm laser (compared to the 405 nm laser) requires an increase in excitation duration [46]. Phototoxicity from high intensity laser exposure is a limiting experimental factor [66]. Dead cells can be identified and avoided by staining with a live/dead stain during FACS sorting (Fig 5A). Similarly, since Dendra2 can be photoconverted while simultaneously visualizing the green pre-converted fluorophore at 488 nm, it is important that the laser power is reduced when visualizing the Dendra2-green for ROI identification to avoid unwanted photoconversion.

Additionally, optimizing for a conservatively defined ROI reduces off-target and false positive nearby cell photoconversion. To determine the potential of false positive and off-target photoconversion to occur, we performed two troubleshooting experiments (Fig 5B–5D). First, to test whether the reflection of light has the capacity to photoconvert adjacent cells, we applied the 405 nm laser line at varying intensities to an empty ROI surrounded by cells and then performed FACS to determine percent of photoconverted cells (Fig 5B). These experiments resulted in false positive photoconversion only when 50% laser line intensity was used, well above the range of photoconversion intensity values used in our system (5–15%) (Fig 5B, Table 4). Next, we tested the impact of multiple rounds of photoconversion on nearby, non-photoconverted cells within a single field-of-view by measuring their Dendra2-red emission after photoconverting 10 or greater adjacent cells (Fig 5C and 5D). This experiment was conducted in 2D and 3D culture conditions utilizing varying degrees of laser line intensities (Fig 5C and 5D). After rounds of photoconversion under 3D culture conditions within a single field of view, we visualized no significant change in the nearby cells’ red emission with 10% 405 nm laser intensity conditions and little to no change with 20% laser intensity (Fig 5C). However, when utilizing the 405 nm laser at 50% intensity, the Dendra2-red fluorescent signal of nearby cells increased drastically and resulted in no significant difference when compared to the red fluorescent signal of photoconverted adjacent cells (Fig 5C). These data suggest that photoconversion at 50% 405 nm laser line intensity under 3D conditions can lead to the collection of falsely photoconverted cells, unlike cells photoconverted at 10% or 20% laser line intensities. Interestingly, after rounds of photoconversion under 2D conditions, we observed no significant change in the nearby cells’ Dendra2-red emission when utilizing 10%, 20%, or 50% laser line intensities (Fig 5D). It is important to note that the percent laser intensity will vary by microscope and/or system conditions, therefore, intensity values should be determined independently. Overall, the optimal approach is to utilize the lowest laser intensity and exposure time that can successfully photoconvert cells for efficient live cell sorting.

**Table 4:** Photoconversion time course guidelines.

These criteria were established after extensive screening of each cell line and culturing condition. Similar screening should be done prior to establishing a photoconversion regimen for other experimental conditions.			
**Procedure**—Timing 1–4 h			
1. Open the 405 nm shutter and adjust laser power to respective intensity dependent on experimental conditions (see below). Laser intensity may vary by experiment or microscope.			
2. Turn down all other laser lines to zero as they will not be in use during photoconversion.			
3. Set the number of prebleach, bleach and postbleach intervals in the time course frame. Of note, these settings are dependent on experimental conditions and can be enhanced for optimization.			
4. Set ROI and run experiment. Continue as needed until all ROI are photoconverted.			
**Experimental conditions**	Non-adherent	3D spheroid	2D monolayer
405 nm laser intensity for photoconversion	5%	15%	10%
Repetitions	1	1	1
Prebleach interval	1	1	1
Bleach 3–5 sec interaction	1	2	3
Postbleach intervals	1	1	1

#### Fluorescence activated cell sorting

A fluorescence activated cell sorter (FACS) with a minimum requirement of two-color flow cytometry is used to isolate user-defined cells based upon fluorescent state. With Dendra2, the green cells include one or more phenotype(s), and the red cells include the photoconverted single or set of cells photoconverted based on phenotype. We typically ensure at least 50 cells or greater are photoconverted to account for cell loss due to viability or, in the case of 3D samples, matrix degradation and cell retrieval steps. For 3D collective invasion experiments, multiple follower and leader cells were separately photoconverted, harvested from their ECM and sorted for downstream applications. In all experiments, negative controls were used to set initial gating: Dendra2 positive cells not exposed to 405- or 488 nm laser lines to determine autofluorescent signal emission in the red channel (yellow green laser line (YG), bandpass filter: 582/15 Dendra2-red), live/dead cell staining to collect only live cells and avoid dead cell autofluorescence, and Dendra2 cells exposed to 405 nm laser line, but purposely not photoconverted to determine percent of cells falsely photoconverted (these cells were exposed to the same photoconversion time course as the positive control) (Fig 5A). Non-photoconverted cells were detected for emission within the Dendra2-red channel to determine the rate of false positives, and photoconverted cells were detected and gated for sorting within the red channel (Fig 5A). FACS gating for the collection of photoconverted cells relied on the detection of separate events (Fig 5A). Sorted populations are typically 95% or more in fluorescent purity; however, samples may include false positives. To ensure FACS purity, a 100-fold separation between non-photoconverted (based upon gating parameters set by the negative control) and photoconverted (Dendra2-red positive) cells is desired (Fig 5A). Photoconversion parameters can be altered to optimize sorting efficiency. Similarly, we recommend performing a trial experiment where a defined number of cells are photoconverted and sorted to test SaGA platform efficiency. After sorting, cells for further phenotypic analysis were replated into complete growth medium for long-term cellular cultivation and propagation. Cells for immediate multi-omic profiling were pelleted, flash frozen, and then stored in a negative 80 °C freezer.

After utilizing FACS to isolate Dendra2-red photoconverted cells, there are a variety of possibilities for downstream analysis to determine the molecular significance of the phenotypically isolated cell subpopulation. Depending upon the users’ experimental design and phenotypic subpopulation of interest, the number of photoconverted cells can vary. For 3D collective invasion SaGA experiments, rare subpopulations (with low cell count) were successfully isolated and analyzed to be epigenetically, transcriptionally, and metabolically heterogeneous [27–31]. The number of photoconverted cells is at user discretion and can be adjusted to fit the experimental parameters. Multiple sequencing techniques are becoming readily available to produce analyses from low cell inputs [67–69]. These approaches provide novel sequencing feasibility to broadly define mechanistic cellular differences within smaller subpopulations. Similarly, single cell sequencing techniques can be utilized to determine whether the cells of interest contain additional heterogeneity, by immediately dropping photoconverted single cells into a multi-well plate (Table 1, Fig 2F and 2G). Cell subpopulations can also be submitted for bulk multi-omic analysis like RNA sequencing or DNA methylation array (Table 1). Likewise, to determine whether the isolated population(s) maintains its respective phenotype over a series of passages (i.e., phenotypic stability), cells can be cultured for further downstream analysis. Together, these downstream analyses provide multi-dimensional molecular depth to the phenotypic distinctions.

#### Comparison with other methods

Cell subpopulation heterogeneity can be assessed using several approaches, including flow cytometry analysis, live-cell imaging, and single-cell multi-omics. However, these methods have limitations since they are unable to directly link live cellular phenotypes, geographic information, and molecular signatures. The SaGA platform leverages multiple experimental modalities to generate multi-scale datasets that integrates molecular, phenotypic, and spatial data.

FACS is a technique initially developed for immune cell classification, and now is utilized in a variety of fields to identify subpopulation heterogeneity within a larger population [70–72]. Traditional approaches utilizing FACS often isolate subpopulations with known markers, making it difficult to identify novel subpopulations utilizing FACS alone. SaGA takes advantage of the ability of FACS to isolate fluorescent single cells and couples that with the preservation of historical spatial and phenotypic information. Importantly, SaGA combines live-cell imaging with FACS to enable propagation of the isolated cells, which we have shown can maintain stable phenotypes over time [27].

These spatially defined and isolated cells can be further analyzed by multi-omics to evaluate molecular signatures and define novel subpopulations. For example, single-cell RNA sequencing has led to the resolution of small, rare subpopulations within the bulk population [73]. However, the power of this approach is limited by the collection process of the cells. Spatial localization is lost upon dissociation of cells for single-cell sequencing. Consequently, single-cell sequencing data alone does not provide insight to phenotypic or spatiotemporal distinctions within the established subpopulations. Since SaGA allows for the precise isolation of cells based on phenotype of interest, it adds context to the datasets driven by multi-omic platforms. By using a photoconvertible approach for cell selection, SaGA can be applied to many different contexts, depending on the interest of the researcher. This is an advantage over selecting cells using microfluidic systems. While the field of microfluidics has evolved to incorporate several methods to distinguish cells and isolate cell subpopulations [74, 75], these techniques are still limited in their capacity to maintain spatial information, as cells are applied to these systems in suspension. It would be difficult to isolate adherent cells of interest or cells invading from a spheroid using a standard microfluidic, in contrast to SaGA [27]. Additionally, SaGA can be performed using equipment commonly available to researchers in the biomedical field without requiring expertise to engineer the microfluidic.

The recent advancements of spatial multi-omic methodologies allow for comprehensive assessment of molecular phenotypes in tissue while retaining spatial tissue context [17, 76–82]. For example, spatial transcriptomics can assess global gene expression patterns and integrate these data with positional localization of cells [80, 83, 84]. While these methods identify different cell populations within a single tissue and maintain the tissue’s architecture, the samples are frozen or fixed. SaGA is advantageous in that the samples are viable throughout the entire process and a historical live cell phenotype can be integrated. Cells are identified and isolated during live-cell microscopy and the purified population can be grown for long-term cultivation with traditional tissue culture techniques. This allows for either immediate sequencing analysis or analysis after long-term propagation. The populations isolated by SaGA can be analyzed by a myriad of live-cell assays depending on the user’s interest, not limited by experimental conditions (i.e., fixed tissue or in suspension).

## Materials and methods

The protocol described in this peer-reviewed article is published on protocols.io, https://dx.doi.org/10.17504/protocols.io.14egn34yml5d/v1 and is included for printing as S1 File with this article.

## Expected results

The SaGA platform affords the unique ability to isolate live cells based upon image-able whole cell or organelle morphological distinctions. The initial technical setup for SaGA, such as introduction of photoconvertible tags or determining imaging parameters, may require initial troubleshooting. However, once established, live cell spatiotemporal multi-omic analysis can be performed utilizing the same experimental parameters.

### Cell transfection and transduction

Dendra2 can be engineered onto a variety of protein targets. Importantly, introducing exogeneous tags to a protein can result in altered protein function and/or activity, and potentially feed forward within the experimental system resulting in cellular, population and subpopulation behavioral changes. To this end, with both the H2B-Dendra2 and pal-Dendra2 PCFPs, we experimentally confirmed that our cellular attributes and phenotypes of interest (proliferation, cell-cell junctional integrity, biomarker expression, spheroid formation, collective invasion) were maintained upon addition of Dendra2 (Fig 3). Similarly, when applying the SaGA platform to alternate research questions, we recommend performing similar experimental comparisons to probe the phenotype of interest with and without the protein tag.

### Live cell imaging and photoconversion

Isolating viable cells based upon a phenotype of interest requires defining an experimental window in which the phenotype occurs. For example, after monitoring H1299 3D spheroid invasion, we determined that day 5 presented clear leaders and followers within the collective invasion pack that can be photoconverted (Fig 2). This time course will vary depending on experimental design. Most scanning confocal microscopes are equipped with scanning-FRAP modes that can be adapted to photoconvert live cells within a specific region of interest.

### Fluorescence activated cell sorting

FACS is optimally performed when at least 50 cells or greater are photoconverted for sorting to account for cell loss during the cell preparation, selection, and isolation (Steps 39–56). The inclusion of a live/dead stain aids in the sorting of viable cells and to assess any death due to laser intensity or 3D degradation mechanisms. To enhance the number of live photoconverted cells during FACS, we recruited colleagues to streamline isolation steps. One person was designated to either photoconvert (Steps 30–40), prepare cells for FACS (Steps 41–56), or sort cells for analysis (Steps 57–69). Utilizing this methodology, we were able to photoconvert and sort 100s of live cells in one work day for downstream analysis.

### Downstream application

Utilizing SaGA-derived leader and follower cells, we performed multi-omic analysis to extrapolate DNA methylation status and bulk transcriptomic profiling [27, 29–31]. We established that leaders and followers maintain stable differences at the epigenetic, genetic, metabolomic, and transcriptomic level [27–31]. Similarly, propagation of isolated leader and follower subpopulations generate stable phenotypes for over 30 passages (Fig 2F) [27]. Together, these data showcase the molecular significance in phenotypic positioning. We envision that SaGA can be applied to a broad range of experimental studies to further exploit distinct cellular responses within a parental population, thereby continuing to identify critical cell subpopulations and their mechanistic dependencies.

### Additional notes

Additional troubleshooting notes can be found in Table 5.

**Table 5:** Troubleshooting table.

Step	Problem	Possible reason	Solution
18	Cells are unable to form spheroid	Low cell—cell adherence junction expression, low incubation time	Repeat centrifugation (step 19) and/or incubate for an additional 24 h.
33	Cells are shrinking; detaching from plate; swelling	Inadequate cell culture conditions on tabletop incubator	Ensure that the incubator is working at the appropriate temperature, pressure, and CO2 level.
35	Poor imaging resolution	Scanning pixel size and/or line averaging amount is too low	Increase these imaging acquisition parameters to increase resolution.
37c	No fluorescent signal in the red channel	Laser intensity value is too low resulting in low to no photoconversion	Increase number of repetitions and/or bleach iterations. May need to increase laser intensity.
37c	No fluorescent signal in the red channel	Laser intensity value is too high resulting in photobleaching ROI	Disregard ROI, decrease laser intensity, and select another ROI.
37c	Cell shrinking or swelling after photoconversion	Laser intensity too high resulting in phototoxicity	Disregard ROI, decrease laser intensity, and select another ROI.
37d	Low fluorescence in the red channel	Low photoconversion efficiency	Increase number of repetitions and/or bleach iterations. May need to increase laser intensity.
44,45	Unable to degrade matrix	Enzyme concentration too low, inadequate incubation time	Increase enzyme concentration or incubation time. Agitate matrix with pipette tip more frequently to encourage degradation.
47	Unable to degrade cell-cell junctions within spheroid	Enzyme volume or concentration too low, inadequate incubation time	Increase concentration, volume, or incubation time. Gently vortex to encourage junction cleavage.
68	Number of cells recovered is higher than number of photoconverted cells	Off-target photoconversion due to inadequate ROI placement and/or autofluorescence	Create a stricter ROI to ensure no off target or false positive photoconversion of nearby cells. Some cell types emit autofluorescence, ensure cytometer voltage settings are set to allow for enough separation between those autofluorescent cells and those that were photoconverted. Decrease laser intensity or time course on microscope to reduce off target photoconversion.
68	Low cell viability post FACS	Inadequate sample preparation and/or maintenance	Keep cells on ice to slow intracellular metabolism and increase survival. Avoid generating a dry pellet or air bubbles during processing. Air bubbles may create a surface tension that is toxic to the cells. Avoid vigorous vortexing and instead mix with gentle pipetting. If cell centrifugation is necessary post FACS, apply low speeds (125–250 g RT).
72	Poor cell proliferation and propagation	Poor collection conditions; not enough cells; crucial growth factors not present	Sort into culture media with at least 20% FBS to increase growth factors and promote cell survival. Coat cultivation plates with protein to promote cell adhesion. Plate cells on smaller surface area plate to facilitate cell—cell communication to promote cell survival.

## Supporting information

S1 File Step-by-step protocol, also available on protocols.io. (PDF)
