# Supplementary material for: A live-cell platform to isolate phenotypically defined subpopulations for spatial multi-omic profiling
Source: PLoS One. 2023 Oct 11;18(10):e0292554. doi: 10.1371/journal.pone.0292554 (PMC10566726; doi:10.1371/journal.pone.0292554)
Supplement: S1 File — (PDF) [file pone.0292554.s001.pdf]

A live-cell platform to isolate phenotypically defined subpopulations for spatial multi-omic profiling

Tala O. Khatib[1,2,3,7], Angelica M. Amanso[1,2,7], Christina M. Knippler[1,2], Brian Pedro[4], Emily R. Summerbell[5], Najdat M. Zohbi[6], Jessica M. Konen[1,2], Janna K. Mouw[1,2], Adam I. Marcus[1,2,3]

[1]Department of Hematology and Medical Oncology, Emory University School of Medicine, Atlanta, Georgia, USA

[2]Winship Cancer Institute of Emory University, Atlanta, Georgia, USA

[3]Graduate Program in Biochemistry, Cell, and Developmental Biology, Emory University, Atlanta, Georgia, USA

[4]Department of Pathology, Johns Hopkins School of Medicine, Baltimore, Maryland, USA

[5]Office of Intramural Training and Education, The National Institutes of Health, Bethesda, Maryland, USA

[6]Graduate Medical Education, Piedmont Macon Medical, Macon, Georgia, USA

[7]These authors contributed equally

SHORT ABSTRACT

In this technique termed **S**p**a**tiotemporal **G**enomic and Cellular **A**nalysis, we exploit phenotypic and image-able variations within a parental cell population to isolate and characterize distinct cellular subpopulations. Our protocol begins with the preparation of cells in non-adherent, 2D, and 3D *in vitro* environments (Steps 1 – 29). Next, we select and isolate phenotypically distinct cell(s) utilizing live confocal microscopy and fluorescence activated cell sorting (Steps 30 – 69). Lastly, we provide multi-omic or phenotypic modes of analyses for the SaGA-isolated subpopulations to determine unique biological phenomena (Steps 70 – 72).

KEYWORDS

Cellular heterogeneity, fluorescence activated cell sorting (FACS), confocal microscopy, cellular isolation, tissue culture, cell selection, intercellular heterogeneity, intratumoral heterogeneity

MATERIALS

Reagents

Biological

- H1299 and RPMI8226 cell lines purchased from American Type Culture Collection, cat. nos. CRL5803 and CCL-155, respectively.
- Lenti-X 293T cells purchased from Clontech Labs cat no. 632180.

  Warning: Authenticity of cell lines should be validated via STR profiling. Sterility of cell lines should be maintained by appropriate and routine checks; these include morphological checks by microscopy, and mycoplasma contamination checks by Myco Alert Mycoplasma Detection Kit (or similar).

Technical

- Accudrop Beads (BD Biosciences cat. no. 345249)
- Annexin V, Pacific Blue Conjugate (Thermo Scientific cat. no. A35122)
- Annexin V, Alexa Fluor 680 (Thermo Scientific cat. no. A35109)
- Collagen I, High Concentration, Rat Tail, 100 mg (Corning cat. no. 354249)
- Collagenase/Dispase Roche (Millipore Sigma cat. no. 11097113001)
- CS&T Research Beads (BD Biosciences cat. no. 650621)
- DPBS, no calcium, no magnesium (Gibco cat. no. 14190250)
- Fetal Bovine Serum, Regular, USDA Approved (Corning cat. no. 35-011-CV)
- Fetal Bovine Serum, Dialyzed, US Origin (Thermo Fisher Scientific cat. no. 26400044)
- Growth Factor Reduced Basement Membrane Matrix (Corning cat. no. 356231)
- Lipofectamine 3000 Transfection Reagent (Invitrogen cat. no. L3000001)
- MycoAlert Mycoplasma Detection Kit (Lonza cat. no. LT07-118)
- OptiMEM I (Gibco cat. no. 31985062)
- Palmitoylated Dendra2, (Emory University, Gary Bassell Lab)
- Penicillin Streptomycin (10,000 U/mL) (Thermo Fisher Scientific cat. no. 15-140-122)
- pLenti.CAG.H2B-Dendra2.W Plasmid (Addgene cat. no. 51005)
- pMD2.G (Addgene cat. no. 12259)
- Polybrene (Millipore cat. no. TR 1003-G)
- psPAX2 (Addgene cat. no. 12260)
- RPMI 1640 [-] L-glutamine, Phenol Red (Gibco cat. no. 11875101)
- RPMI 1640 [-] L-glutamine Medium, no Phenol Red (Gibco cat. no. 11835030)
- Sterilized Distilled Water
- Trypan Blue Solution 0.4% (Sigma-Aldrich cat. no. T8154)
- Trypsin/EDTA Solution (Lonza cat. no. CC-5012)
- Trypsin Neutralizing Solution (Lonza cat. no. CC-5002)

Reagent Set Up

- Complete medium – Prepare RPMI 1640 (with and without phenol red) supplemented with 10 % (vol/vol) FBS and 1 % (vol/vol) Penicillin Streptomycin. Prepared media can be stored at 4 ^{o}C for two weeks. Maintaining sterile culture, remove necessary volume as needed and pre-warm aliquot to 37 ^{o}C before use (unless otherwise noted).
- Flow buffer – Prepare fresh on the day of use by supplementing RPMI 1640 or appropriate base media (without phenol red) with 5 % (vol/vol) dialyzed FBS. Alternatively, calcium free reagents can be used to reduce cell aggregation.

3D matrix embedment experiments

Important: Particularly critical for epithelial and endothelial cell differentiation and function, the reconstituted basement membrane (rBM) provides a critical structural interface between certain cell types and their surrounding environment, acting as a mechanical buffer and barrier to both cellular and molecular traffic (1, 2). rBM derived from Englebreth-Holm-Swarm murine tumors provides a laminin and type IV collagen rich microenvironment conducive to studying 3D cell growth and differentiation, as well as morphogenesis (breast, lung) and invasion. As such, rBM provides a microenvironment conducive to collective invasion for many of our lung tumor cell lines (Fig. 2) (3, 4). Conversely, for our studies using the 4T1 murine model of breast cancer, we find that fibrillar type I collagen provides a microenvironment supportive of multicellular pack invasion for the largely e-cadherin positive 4T1 cells (data not shown).

- Recombinant basement membrane master mix – Thaw growth factor reduced recombinant basement membrane (often referred to as its tradename Matrigel, rBM) at 4 ^{o}C overnight and maintain on ice to avoid polymerization at room temperature. Spheroids are embedded in 5 mg/mL rBM (in full media) and then plated onto a glass bottom dish. Note, the polymerization characteristics of rBM may change from lot to lot of Matrigel; as such, the concentration may need to be adjusted on a lot-specific basis. Additionally, cell secreted growth factors and cytokines will vary between lots of non-growth factor depleted Matrigel; growth factor reduced Matrigel leads to a more consistent composition (5).
- Collagen I master mix – Keep stock collagen I (high concentration derived from rat tail) solution at 4°C or on ice until ready for use. On ice, supplement stock collagen I with 10 % (vol/vol) PBS to a working concentration of 3 mg/mL. Check pH of solution with pH strips and adjust pH to approximately 7.0. Keeping working solution on ice until ready to use. Do not store dilute solution long-term.

- Collagenase/dispase cocktail: Suspend 100 mg of collagenase/dispase (C/D) in 1 mL of PBS or base media (without added growth factors or FBS) for a 100 mg/mL stock solution. Importantly, calcium is an important factor for enzymatic stability and activity in this cocktail; therefore, in the case of PBS, calcium supplemented PBS is required. Pipette gently to thoroughly resuspend. Sterilize with a 0.2 μm filter. Make aliquots of stock at 50-100 μL per aliquot. Freeze at -20 ^oC until ready for use.

Equipment

Tissue culture

- CellDrop Automated Cell Counter (DeNovix cat. no. CellDrop FL-UMLTD)
- CO_2 Incubator Forma Series II Water Jacketed (Thermo Fisher Scientific cat. no. 3110)
- Cryostorage Container, Locator 4 Plus (Thermo Fisher Scientific cat. no. CY509108)
- Eppendorf Centrifuge 5425 (Thermo Fisher Scientific cat. no. 13864455)
- Eppendorf Centrifuge 5810/5810R (Millipore Sigma cat. no. EP022628168)
- Falcon Round-Bottom Tubes with Cell Strainer Cap, 5 mL (Stem Cell Technologies cat. no. 38030)
- Isotemp Dual Digital Water Bath (Fisher Scientific cat. no. FS-215)
- Sterilized Biosafety Cabinet (Labconco cat. no. 3440009)
- Ultra-Clear Microcentrifuge Tube 1.7 mL (DOT Scientific cat. no. 609-GMT)
- Ultra-Low Attachment Multiwell Plates, Sterile (Corning cat. no. 29443-034)
- μ-Slide 8-Well Glass Bottom (Ibidi cat. no. 80827)
- 15 mL Conical Centrifuge Tube (VWR, cat. no. 430052)
- 35 mm Glass Bottom Dish (MatTek, part no. P35G-1.5-14-C)
- 50 mL Conical Tube (VWR cat. no. 430290)
- 75 cm^2 U-shape cell culture flask, canted neck (Corning cat. no. CLS430641U)

Imaging and FACS

- BD FACS Aria II Cell Sorter
- Inverted Microscope, Olympus CKX41
- Leica TCS SP8 Inverted Point Scanning Confocal equipped with Galvano and 8 kHz resonant scanners, Tokai Hit stage top incubator for CO2 and temperature control, two multi-alkali PMTs, two HyDs, and one transmitted light PMT

Software

- BD FACSDiva Software

- FlowJo
- Graphpad Prism
- ImageJ or FIJI
- LAS X Imaging Software

PROCEDURE

Sample Preparation

Guidelines**:** This section describes our approach to handling a variety of cell lines and patient samples in culture. Non-adherent and 2D conditions are respective to those particular and potentially uniquely appropriate to the experimental questions and cell treatments. 3D tumor spheroid formation is described in detail by using the example human NSCLC H1299 cell line. See Table 2 for parameters to consider for optimizing the 3D invasion assay procedure to other cell lines and systems.

Thawing and maintenance of cells

Timing 1 h (thawing cells), 1 week (cell growth and maintenance)

1. Prepare cell culture media as described in reagent set up.
2. Warm desired volume of culture media in 50 mL conical tubes at 37 °C in bead or water bath.
3. Prepare tissue culture appropriate laminar flow hood using UV light and wipe down all working surfaces with 70 % ethanol (EtOH, vol/vol). Perform all cell culture within the laminar flow hood to maintain sterility. Use rigorous aseptic/sterile tissue culture technique where appropriate.
4. Rapidly thaw a vial of RPMI8226-Dendra2 or H1299-Dendra2 (either pal- or H2B) cells by gentle agitation in 37 °C water bath.
5. Decontaminate the vial by spraying with 70 % (vol/vol) EtOH.
6. In the tissue culture hood, quickly open and transfer the vial contents to a 15 mL centrifuge tube containing 9.0 mL complete culture medium and spin at 125 g at room temperature (RT) for 5 min to pellet the cells out of solution. Speed and times may vary between cell lines.
7. Carefully remove the supernatant from above the pellet, taking care not to disturb the pellet. Resuspend the cell pellet with complete media and dispense into a 25 cm^2 or a 75 cm^2 (if working with cell concentrations greater than 1 x 10^6) culture flask. The density of the cells and volume of culture media may vary between cell lines.

8. Incubate at 37 °C and atmosphere of 95 % Air, 5 % CO_2. These conditions may vary by cell line.
9. Optional: when necessary, change media after 24 h to eliminate cells that do not survive thaw cycle.
10. Allow cells to recover from thaw (typically 2-3 days) and passage 1X prior to experiment set up.
11. Ensure media renewal occurs once every two days, or as appropriate for the cell line.
12. Passage cells at less than 70 % confluency and ensure to collect all cells.

    <u>Important step:</u> With heterogenous cell lines and patient samples, under normally adherent conditions, floating cells may be viable, non-adherent subpopulations within the greater population. When necessary, collect the floating cells, as well as the difficult to de-adhere cells, to ensure that the overall population doesn't artificially drift in subpopulation composition.

    a) For adherent cell passaging:
        i. Collect conditioned/spent media in 50 mL conical tube.
        ii. Wash 1X with PBS and add to the conditioned media.
        iii. Detach cells with method of choice (trypsin, EDTA, Accutase). Different cell types may require distinct cell detachment mechanisms; therefore, it is best to determine the best passaging conditions for your system.
        iv. Neutralize trypsin enzyme with an appropriate trypsin neutralizing solution (TNS) at a 1:1 trypsin to TNS ratio. While some TNS product utilize serum (FBS or otherwise) to provide excess substrate for the trypsin to enzymatically digest protein molecules, an alternative – particularly for cells cultured serum free – is the use of a TNS product consisting of a 1X PBS solution containing 0.0125 % (vol/vol) soybean trypsin inhibitor.
        v. Pipet additional complete media onto plate to wash and collect all cells from plate and add to the 50 mL conical tube.
        vi. Centrifuge conical tube at 125-250 g RT for 5 min, centrifuge speed depends on the cell line.
        vii. Resuspend in 3 mL of complete media.
        viii. Count cells using 0.4 % Trypan blue solution at a 1:1 ratio with hemocytometer.

b) Non-adherent cells do not require enzymatic cleavage from cell plate and can simply be collected from plate at time of experimentation. Cell concentrations may vary by plate or cell size.

Option A: Non-adherent sample preparation for photoconversion

Timing 1 h (cell collection, counting and plating)

13. Collect and count RPMI8226-Dendra2 cells using 0.4 % Trypan blue at a 1:1 ratio with hemocytometer.
14. 0.5×10^6 cells are isolated from the bulk sample and spun down at 125 – 250 g RT for 5 min, centrifuge speeds may vary by cell line. Number of cells will vary by plate and cell size.
15. Resuspend cells in 200 µL phenol-red free complete media and transfer to 8-well glass bottom slide chamber. Cell number is dependent on cell morphology and well dimensions. Important step: Cells in suspension will gravitate down to the bottom of the well over time. To avoid cell plane variability and maintain a relatively constant z – plane when imaging, incubate the cells at least 30 min in a static environment prior to photoconversion. These cells will continue to move across the X, Y and Z planes over time and microscope parameters should be adjusted accordingly.

Option B: 3D cell culture sample preparation for photoconversion

Timing: 1 h (cell plating), 72 h (incubation), 2 h (embedding into matrix), 24 – 48 h (incubation for invasion overtime)

Important: During processes such as morphogenesis and tumor progression, a 3D microenvironment provides a physical and biochemical scaffold for cells to grow, self-organize and remodel surrounding pericellular and extracellular matrices. *In vivo* and *in situ*, 3D matrix microenvironments are comprised of cellular (fibroblasts, lymphocytes, endothelial cells, etc.) and non-cellular (ECM, cytokines, growth factors) components that actively mediate cell migration and invasion, proliferative capacity, and differentiation (6-8). Distinct ECMs are highly specialized and organized networks integrating a myriad of molecules that establish an essential architectural support structure for cells, tissues, and organs along with providing a substrate for cell adhesion and traction (2, 9). Various 3D *in vitro* systems utilize reconstituted ECMs to assess growth, motility, and invasive phenotypes. Depending on cell type, biological activity of choice (such as single cell and collective invasion), different ECMs may be better suited to asking specific biological questions.

16. To generate spheroids, plate 3,000 H1299 cells in 200 µL (1.5×10^4 cells per mL) in a low adherence 96-well plate. Create 18 spheroids per condition to embed 10 – 12 spheroids in matrix. Excess spheroids are created to account for shape and stability deformities during incubation period for spheroid formation (Table 2). The cell number may be adjusted for different cell lines and spheroids of different diameters (Table 2).
17. Centrifuge plate at 450 g RT for 10 min to collect cells at the center of each low absorbance round-bottom well.
18. Incubate cells for 72 h for cell – cell junction formation within the spheroid.

    Important step: Some cells may resist adhering to one another to form spheroid. Incubation time varies on the cell line and in some instances, during the 72 h spheroid formation period, a once a day, 10 min at 450 g RT spin may encourage spheroid formation (Table 2). *Additional note*
19. Prior to the day of embedment, snip an experimentally appropriate number of 1,000 µL and 200 µL pipet tips for embedding spheroids into matrix. Autoclave to re-sterilize tips and allow to cool to room temperature.
20. A standard light microscope can be used prior to spheroid removal from the round bottom plate to validate spheroid integrity has been maintained (no debris contaminating or altering spheroid) (Table 2). Depending on the cell line, spheroids may not be spherical in shape. While sphericity is not necessary for transfer, spheroid stability is necessary for extraction from the round bottom plates and successful embedment. Non-stable spheroids/structures may fall apart during the process.
21. Collect usable spheroids with tip-snipped 1,000 µL pipet tips into 1.7 mL microcentrifuge tube - one experimental group per tube. The same snipped-tip can be used for all spheroids within the same experimental group. Pipet 8 spheroids per 1.7 mL microcentrifuge tube. The number of spheroids included can be increased or decreased depending on the experiment.
22. Allow the spheroids to sink to the bottom of the microcentrifuge tube. Remove excess media taking care not to pipet up the spheroids. Remove single and detached cells by washing spheroids with 1 mL of complete medium 2X by pipetting media along the edges of the tube in a circular motion, allowing the spheroids to sink, and removing excess media.

    Important step: Treat spheroids with care. Avoid shaking tube, do not vortex. Spheroids are large and solid enough to visualize as they drop to the bottom of the microcentrifuge tube.

23. Collect spheroids in 100 µL 3D master mix using tip-snipped 200 µL pipetmen and plate in 35-mm glass bottom dish.
24. Use a second unsnipped 200 µL pipette tip to carefully spread master mix to cover entire glass surface.
25. With a third unsnipped 200 µL pipette tip, carefully move and spread spheroids for equal distribution.

    Important step: If there is an air bubble in matrix, 70 % EtOH (vol/vol) can be used to eliminate the air bubble. Dip a clean 200 µL pipette tip into ethanol and poke the bubble. The alcohol breaks the surface tension of the matrix, causing the bubble to pop.
26. Allow >30 min for matrix to polymerize. After complete polymerization, add 1.5 mL pre-warmed complete medium and incubate. Note that the addition of cold media to the temperature-sensitive polymerized rBM may destabilize the matrix, leading to the loss of matrix and spheroids from the surface into the media.
27. Image every 24 h if monitoring surface area or circularity (Table 3).

Option C: 2D cell culture sample preparation for photoconversion

Timing: 1 h (cell collection, counting and plating)

28. Passage cells at < 70 % confluency and plate cells on glass bottom plate for photoconversion the following day. Cell number will be dependent on plate size and cell morphology. Cell confluency will be dependent on experimental question.
29. If distinct phenotypes are present temporally upon treatment with drug or other additive factor, adjust time course and confluency to match these criteria.

Confocal imaging and photoconversion

Important: This section describes the set-up and application of the Leica TCS SP8 inverted scanning confocal microscope for photoconversion during live cell imaging. We discuss a few Leica systematic anomalies; however, all steps can be adapted to fit the technical set up for most scanning confocal microscopes. The protocol by Chudakov, D.M., *et al*. provides specifics on Dendra2 photoconversion utilizing either the 405 nm or 488 nm laser lines and can be referenced for more detail (10). The protocol we describe here uses the 405 nm laser line.

Microscope and laser set-up

Timing: 2 h (stage top incubator equilibration), 0.5 h (cells equilibrating to stage top incubator conditions)

30. Prepare stage top incubator to maintain standard tissue culture conditions, typically 5 % CO_2 at 37 °C. These conditions may vary by cell line and are utilized to facilitate typical cellular phenotypes under defined experimental conditions.
31. Fill stage top incubator with enough autoclaved distilled water to maintain humidity and compensate for evaporation during imaging. Allow the incubator to equilibrate for at least 2 hours.
32. Turn on the computer, microscope, scanner, and laser power source. Photoconversion requires three laser lines: 405 nm for photoconversion, 488 nm for visualization of Dendra2-green (excitation and emission peaks: 490/507 nm), and 561 nm for visualization of Dendra2-red (excitation and emission peaks: 557/573 nm). Alternatively, can use 543- or 568 nm laser lines in place of 561 nm for visualization of Dendra2-red (emission spectral ranges: 570-670 nm).
33. Place the plate with cells inside stage top incubator and incubate for 30 min to allow cells to equilibrate to microscope conditions. *Additional note*
34. Open laser configuration window and turn on the Diode 405 nm (UV) laser, Argon laser (visible), and the DPSS 561 nm laser lines.
    <u>Important:</u> Argon laser power intensity settings are specific to laser line and microscope conditions.
35. Image acquisition set-up.
    a) Turn off resonant scanning. Resonant scanning is a mode to increase imaging speed by gathering images at a rate of 30 frames per second or higher. This is typically used for overnight live cell imaging and therefore is not required for photoconversion.
    b) Set the scanning parameters to the XYT (XY Time) mode. Time is necessary for photoconversion (Step 37d). Z-plane is not required for photoconversion because high intensity laser exposure reaches multiple planes within the defined region of interest (ROI).
    c) Set scanning pixel size to be 1024 x 1024 or lower. Resolution can be sacrificed for increased imaging speed and pixel dwelling time within the ROI. High resolution is not required for successful photoconversion.
    d) Use the default scanning speed (400 – 800 Hz). This is the acquisition rate of pixels per second.

e) Determine the zoom factor empirically. The zoom is dependent on the objective lens and user preference. A typical starting point is a zoom factor of 2 and adjust as needed based on ROI.

f) Set line averaging at 2 and change as needed depending upon resolution needs or scanning speed requirements.

Important step: Scanning pixel size, speed, zoom factor, and line averaging are ultimately dependent on the user's phenotype of interest. For example, isolating cells based on their positional phenotype within the population can be captured at a lower resolution. Isolating cells based on organelle level distinctions (such as differences in mitochondria localization) can require higher resolution. *Additional note*

36. Configure laser parameters.

Warning: The two options for configuring multichannel imaging are (i) simultaneous or (ii) sequential scanning. Simultaneous scanning images every channel at the same time. Sequential scanning images each channel independently and can switch after every line, frame, or stack. A disadvantage to simultaneous scanning is that overlap in the emission spectra of two dyes will lead to crosstalk and caution should be taken. The Leica LAS X software provides scanning recommendations and can be referenced during set-up.

a) Open the laser configuration window for the 405 nm, 488 nm, and 561 nm laser lines.

b) Set the 488 nm and 561 nm laser lines to sequential scanning to avoid emission spectra crosstalk. Use the 'between line' scanning feature so live cell movement does not lead to signal artifact between channels.

   Important: Short pixel dwelling time during speed scanning (800 Hz) to visualize Dendra2-green localization with 488 nm excitation light, likely will not cause Dendra2 photoconversion.

c) Visualize the sample and adjust the objective to the desired focal plane.

d) Use live view to adjust the detector, gain, and laser intensities for optimal exposure.

Warning: Maintain minimal laser power output for visualizing Dendra2-green and keep in mind that different microscopes will vary in required laser power intensity; however, in all cases too high laser power will result in photobleaching, cell toxicity, and cell death. Phototoxicity can be assessed utilizing common cell viability assays, like Annexin V staining (Step 54).

e) Use the photomultiplier tube (PMT) detector for photoconversion because during the bleach sequence, high intensity illumination targets the region of interest (ROI). The hybrid detector (HyDs) may switch off to protect the ROI from photon overload, resulting in loss of the post-bleach sequence.

Photoconversion

Timing: 1 - 4 h (photoconversion of user-defined and phenotypically distinct cells)

37. The photoconversion parameters set-up utilizing the FRAP interface.

    Important step: Most confocal microscope software is equipped with a Fluorescence Recovery After Photobleaching (FRAP) feature. The FRAP parameters are then suitable for photoconversion.

    a) Turn the zoom-in feature on to allow for precise illumination and ROI selection.
    b) Set the background to zero to ensure that the area outside of the ROI is not exposed to background light. This will decrease the potential for false positive cell photoconversion near the ROI.
    c) Set the 405 nm laser intensity for photoconversion. This is dependent on cell type and experimental conditions. It is important to test various intensities to determine which intensity maintains cell viability while ensuring complete photoconversion for cells within the ROI (Table 4, Fig. 5B – D). *Additional note*

       Warning: Photobleaching occurs when laser intensity for photoconversion is too high and permanently eliminates fluorescent signal within the ROI due to a photon-induced covalent modification. If photobleaching occurs, decrease the 405 nm laser line intensity, and select a new ROI.
    d) Establish a time course for the number of prebleach, bleach, and post-bleach frames. These values are dependent on the experimental design. Optimize the time course by testing various conditions for each sample in study (Table 4). During these screening stages, monitor cells for any unhealthy signs, such as swelling or shrinkage. *Additional note*

38. Select the phenotype-driven ROI and begin the established time course for photoconversion.
39. Repeat steps 37 – 38 as necessary until all user-defined cells are photoconverted.
40. Assess efficiency of photoconversion within the evaluation menu on the user interface (Fig. 4B, C). The integrated density relative fluorescence values pre- and post-photoconversion are shown. The Dendra2-green should significantly decrease and Dendra2-red should significantly increase after photoconversion (Fig. 4B – E).

Important: The H2B-Dendra2 offers more accurate results over pal-Dendra2 because the 405 nm laser line can photoconvert within a singular region (versus around the periphery of the cell) increasing localized excitation and decreasing off-target photoconversion.

Fluorescence-activated cell sorting (FACS)

Important: Due to the different culture conditions that can be used with the SaGA platform, we discuss options for preparing non-adherent cells and samples cultured in 2D and 3D conditions for FACS.

Option A: Non-adherent sample preparation for FACS

Timing: 0.5 h (cell collection)

41. Centrifuge non-adherent cells at 125 – 350 g RT for 5 min. Centrifuge speeds may vary by cell line.
42. Proceed to step 51.

Option B: 3D cell culture sample preparation for FACS

Timing:1.5 h (matrix degradation), 0.5 h (spheroid dissociation)

43. Dilute stock Collagenase/Dispase (C/D) cocktail in sterile media without serum for a working concentration between 1 – 5 mg/mL. For digestion of rBM 1 mg/mL, is sufficient. For digestion of type I collagen, a higher concentration is recommended. Enzyme concentration can be increased if the matrix is difficult to degrade.
44. Option 1: Mince matrix into quarters and place into a microcentrifuge tube with 3 – 4X volume of the minced matrix with the working stock of C/D. Place in a 37 °C incubator. Lightly vortex every 5 – 10 min until matrix is digested.
45. Option 2: Digest the matrix directly in the glass bottom dish. Remove all media prior to adding 3 – 4X volume of the working stock of the C/D digestion buffer. Place dish in a 37 °C incubator. Pipette gently every 5 – 10 min until matrix is digested and cells are released. *Additional note*
46. Centrifuge cells at 150 – 300 g RT for 5 – 10 min
47. Resuspend in trypsin (or similar proteolytic enzyme suitable for cleaving cell – cell junctions) to further digest spheroids and cell clusters into single cells. A standard light microscope can be used to visualize the formation of single cells. *Additional note*
48. Inactivate trypsin with TNS at a 1:1 ratio and centrifuge cells at 125 – 250 g RT for 5 min. Centrifuge speed may vary depending on cell line.
49. Proceed to step 51.

Option C: 2D cell culture sample preparation for FACS

Timing: 1 h (cell collection), 0.5 h (live/dead staining), 0.5 h (FACS cellular preparation)

50. Repeat steps 12.a.i – 12.a.vi for cell collection
51. Wash cells 1X by resuspending the cell pellet in 5 mL of flow buffer.
52. Centrifuge sample for 5 min at 250 g RT to pellet cells.
53. Gently resuspend approximately 1.0×10^6 cells or less into 100 µL flow buffer. For samples with more than 1.0×10^6 cells, resuspend in a larger volume to avoid cell aggregation or flow cytometer clogging.
54. Add 5 µL per 100 µL cell suspension of Annexin V conjugate (per manufacturer recommendations) and incubate for 10 min at RT. Protect samples from light from this point forward.

    Important step: Annexin V conjugate will bind to phosphatidylserine, a marker of an apoptotic cell when exposed on the outer leaflet of the plasma membrane. Marking dead cells in the sample will reduce autofluorescence and increase population resolution to accurately select living, photoconverted cells.
55. Add 200 µL flow buffer per 100 µL solution. Gently pipette to mix and transfer to a 5 mL flow tube with a cell strainer. Place sample on ice for transport to the FACS sorter.
56. Prepare a tube with 1 mL of complete culture medium for collection of cells after FACS. Prior to sorting, run each sample through cell strainer flow tube cap to decrease cell aggregation.

FACS set up

Timing: 1 h (instrument set-up and sterilization)

57. Ensure waste tank is empty and sheath tank is at least 50 % full. Sheath tank should be tightly closed to allow for pressure build up in the system.
58. Power on the computer and activate the compressed airline to at least 80 psi.
59. Switch on the cell sorter and start the sorting software, ensuring the computer has successfully connected to the FACS sorter.
60. Initiate the fluidics start-up and follow the popup tutorial to complete required steps. This start-up will take approximately 10 min.
61. Remove the closing nozzle from the flow cell assembly and attach the appropriate nozzle for your experimentation.

    Important step: Nozzle size is dependent upon cell size. For example, cells that are greater than 25 µm are best suited for a nozzle that is 100 µm or greater in size. Ensure that the software configuration matches the nozzle size of choice.
62. Activate the stream and stabilize to desired flow rate.

63. Calibrate the cytometer with cytometer set-up software and tracking beads. Set drop delay using Accudrop beads. This calibration will take approximately 20 min.
64. Sterilize the cytometer interior with 70 % (vol/vol) EtOH.
65. Sterilize the sample line with 10 % (vol/vol) bleach and rinse with water.

FACS collection

<u>Timing:</u> 1 h (evaluating control samples and isolating photoconverted cells)

66. Use lasers blue (B) 488 nm, yellow/green (YG) 561 nm, red (R) 633 nm, to excite and visualize the following, respectively: Dendra2-green – bandpass filter: B 530/30, Dendra2-red – bandpass filter: YG 582/15, Annexin V (Alexa Fluor 680) – bandpass filter: R 710/50. Additionally, Annexin V pacific blue (laser line: violet 405 nm, bandpass filter: 450/50) can be used in conjugation with Dendra2. Due its low intensity, the 405 nm and 488 nm lasers will not photoconvert Dendra2 while in the FACS sorter.
67. Prepare a workbook to collect live, single cells.
68. Perform the respective controls (as described in experimental design, Fig. 5A) and then flow sort cells positive for Dendra2-red into the collection tube prepared in Step 56 (Fig. 5A). These cells are the phenotypically driven, user-defined cell subpopulation of interest. *Additional note*
69. Process, culture, or store the collected cells based on the analysis of interest (Table 1, Fig. 2, Fig. 6).

Downstream analysis post-FACS

<u>Timing:</u> N/A (dependent upon assay of choice)

70. To sort cells for immediate bulk -omic analysis, collect the same number of cells for each photoconverted population into the desired lysis buffer (such as DNA or RNA lysis buffer) (Table 1).
71. To sort for immediate single cell -omic analysis, sort single photoconverted cells into individual wells of a 96-well plate with desired lysis buffer and immediately place in a negative 80 °C freezer (Table 1).
72. For cell propagation and long-term phenotypic analysis, sort cells into complete growth medium (Table 1). Culture and passage the cells as routinely done for further experimentation. *Additional note*

ADDITIONAL NOTES

Additional troubleshooting notes can be found in Table 5.

Table 1 I Example downstream applications of SaGA-isolated subpopulations			
Experimental approach		Application	Potential outcome
Immediate isolation	*In vitro*	Cell lysis for immediate contents extraction (i.e., protein, RNA, DNA, and ribosomes)	Targeted transient expression profiling via immunoblotting, qPCR, etc.
			Unbiased transient expression profiling via ATACseq, RNAseq, Riboseq, etc.
Long-term cultivation *in vitro*	*In vitro*	Cell behavior, signaling, etc.	Stable phenotype identification, stable subpopulation generation, determination of cooperative phenotype between subpopulations, targeted expression profiling and unbiased multi-omic analysis.
	In vivo	Introduction to model organism	Stable phenotype identification, determination of cooperative phenotype between subpopulations, targeted expression profiling and unbiased multi-omic analysis.

Table 2 I Parameters for spheroid formation			
Parameters	Optimization	Examples	Steps
Seeding density	Spheroid density depends upon cell size, shape, morphology, and rate of proliferation. Various densities should be screened to achieve ~ 500 µm in spheroid diameter upon embedding into matrix. Importantly, the larger the cell number, the larger the spheroid, the greater oxygen differential between the external cells and the cells internal to the 3D structures.	3000 cells/well (H1299, 4T1) 1000 cells/well (A375)	Steps 16 – 18
Nanoparticle contamination	Sterile 96-well plates and/or sterile pipet tips are often contaminated with sterilized nanoparticles that can become embedded within a spheroid and deform its shape. 1.5X spheroids are created to account for unusable spheroids.		Steps 16 – 25
Cell adherence	Heterogeneous cells can express distinct adherence junction profiles to regulate cell – cell junctions and cell – matrix adhesion/interactions. Centrifugating 96-well plate places cells in the center of the well near one another to promote cell – cell junction formation. Upon spheroid formation, different matrices can be screened to determine the ability for cell – matrix adhesion formation and interactions.	rBM (H1299, A375) Collagen I (4T1)	Step 18
Time	After centrifugation, spheroid formation requires a 24 h or more incubation time. Cells can be screened to determine optimal incubation time to maintain both spheroid integrity during the embedding process and cell viability after.	72 h (H1299, 4T1, A375)	Step 18

Table 3 I 3D spheroid invasive area and circularity quantification
It is important to ensure that spheroid invasion dynamics remain largely unaffected when cells are transduced with photoconvertible tag. (The same principles can be applied to confirm no off-target effects from tag in user assay of choice)
**Procedure** – Timing 3 days (imaging and spheroid invasion), 1 h (imaging analysis)
1. Establish and embed spheroids with and without photoconvertible tag (Steps 16 – 26).
2. Image spheroid on day 0, day 1, day 2 using Compound light microscope at 4X (Step 27).
3. Transfer imaging data and open FIJI software (or other software of your choice).
4. Set up analysis tools to determine object circularity and surface area. Use the ‘draw’ to create an outline of each spheroid (including invading cells).
5. Calculate circularity and surface area for each experimental group and export data to excel to determine standard deviation between spheroid technical replicates.
6. Compare results to determine statistically distinct differences in invasive area or circularity between naïve cells and those transduced with photoconvertible tag.

Table 4 I Photoconversion time course guidelines			
These criteria were established after extensive screening of each cell line and culturing condition. Similar screening should be done prior to establishing a photoconversion regimen for other experimental conditions.			
**Procedure** – Timing 1 - 4 h			
1. Open the 405 nm shutter and adjust laser power to respective intensity dependent on experimental conditions (see below). Laser intensity may vary by experiment or microscope.			
2. Turn down all other laser lines to zero as they will not be in use during photoconversion.			
3. Set the number of prebleach, bleach and postbleach intervals in the time course frame. Of note, these settings are dependent on experimental conditions and can be enhanced for optimization.			
4. Set ROI and run experiment. Continue as needed until all ROI are photoconverted.			
**Experimental conditions**	Non-adherent	3D spheroid	2D monolayer
405 nm laser intensity for photoconversion	5%	15%	10%
Repetitions	1	1	1
Prebleach interval	1	1	1
Bleach 3 - 5 sec interaction	1	2	3
Postbleach intervals	1	1	1

Table 5 I Troubleshooting table			
Step	Problem	Possible reason	Solution
18	Cells are unable to form spheroid	Low cell - cell adherence junction expression, low incubation time	Repeat centrifugation (step 19) and/or incubate for an additional 24 h.
33	Cells are shrinking; detaching from plate; swelling	Inadequate cell culture conditions on tabletop incubator	Ensure that the incubator is working at the appropriate temperature, pressure, and CO2 level.
35	Poor imaging resolution	Scanning pixel size and/or line averaging amount is too low	Increase these imaging acquisition parameters to increase resolution.
37c	No fluorescent signal in the red channel	Laser intensity value is too low resulting in low to no photoconversion	Increase number of repetitions and/or bleach iterations. May need to increase laser intensity.
37c	No fluorescent signal in the red channel	Laser intensity value is too high resulting in photobleaching ROI	Disregard ROI, decrease laser intensity, and select another ROI.
37c	Cell shrinking or swelling after photoconversion	Laser intensity too high resulting in phototoxicity	Disregard ROI, decrease laser intensity, and select another ROI.
37d	Low fluorescence in the red channel	Low photoconversion efficiency	Increase number of repetitions and/or bleach iterations. May need to increase laser intensity.
44,45	Unable to degrade matrix	Enzyme concentration too low, inadequate incubation time	Increase enzyme concentration or incubation time. Agitate matrix with pipette tip more frequently to encourage degradation.
47	Unable to degrade cell-cell junctions within spheroid	Enzyme volume or concentration too low, inadequate incubation time	Increase concentration, volume, or incubation time. Gently vortex to encourage junction cleavage.
68	Number of cells recovered is higher than number of photoconverted cells	Off-target photoconversion due to inadequate ROI placement and/or autofluorescence	Create a stricter ROI to ensure no off target or false positive photoconversion of nearby cells. Some cell types emit autofluorescence, ensure cytometer voltage settings are set to allow for enough separation between those autofluorescent cells and those that were photoconverted. Decrease laser intensity or time course on microscope to reduce off target photoconversion.
68	Low cell viability post FACS	Inadequate sample preparation and/or maintenance	Keep cells on ice to slow intracellular metabolism and increase survival. Avoid generating a dry pellet or air bubbles during processing. Air bubbles may create a surface tension that is toxic to the cells. Avoid vigorous vortexing and instead mix with gentle pipetting. If cell centrifugation is necessary post FACS, apply low speeds (125 - 250 g RT).

72	Poor cell proliferation and propagation	Poor collection conditions; not enough cells; crucial growth factors not present	Sort into culture media with at least 20% FBS to increase growth factors and promote cell survival. Coat cultivation plates with protein to promote cell adhesion. Plate cells on smaller surface area plate to facilitate cell - cell communication to promote cell survival.

1. Sodek KL, Brown TJ, Ringuette MJ. Collagen I but not Matrigel matrices provide an MMP-dependent barrier to ovarian cancer cell penetration. BMC Cancer. 2008;8(1):223.
2. Mouw JK, Ou G, Weaver VM. Extracellular matrix assembly: a multiscale deconstruction. Nat Rev Mol Cell Biol. 2014;15(12):771-85.
3. Engbring JA, Kleinman HK. The basement membrane matrix in malignancy. J Pathol. 2003;200(4):465-70.
4. Hapach LA, Mosier JA, Wang W, Reinhart-King CA. Engineered models to parse apart the metastatic cascade. npj Precision Oncology. 2019;3(1):20.
5. Vukicevic S, Kleinman HK, Luyten FP, Roberts AB, Roche NS, Reddi AH. Identification of multiple active growth factors in basement membrane Matrigel suggests caution in interpretation of cellular activity related to extracellular matrix components. Exp Cell Res. 1992;202(1):1-8.
6. Baghban R, Roshangar L, Jahanban-Esfahlan R, Seidi K, Ebrahimi-Kalan A, Jaymand M, et al. Tumor microenvironment complexity and therapeutic implications at a glance. Cell Communication and Signaling. 2020;18(1):59.
7. G. Gritsenko P, Ilina O, Friedl P. Interstitial guidance of cancer invasion. The Journal of Pathology. 2012;226(2):185-99.
8. Haeger A, Krause M, Wolf K, Friedl P. Cell jamming: Collective invasion of mesenchymal tumor cells imposed by tissue confinement. Biochimica et Biophysica Acta (BBA) - General Subjects. 2014;1840(8):2386-95.
9. Frantz C, Stewart KM, Weaver VM. The extracellular matrix at a glance. Journal of cell science. 2010;123(24):4195-200.
10. Chudakov DM, Lukyanov S, Lukyanov KA. Tracking intracellular protein movements using photoswitchable fluorescent proteins PS-CFP2 and Dendra2. Nature Protocols. 2007;2(8):2024-32.
